# Textural and sensory characteristics of sugar‐free biscuit formulated with quinoa flour, isomalt, and maltodextrin

**DOI:** 10.1002/fsn3.2564

**Published:** 2021-09-30

**Authors:** Narges Nadian, Mohammad Hossain Azizi, Hossein Abbastabar Ahangar, Aazam Aarabi

**Affiliations:** ^1^ Department of Food science and Technology Najafabad Branch Islamic Azad University Najafabad Iran; ^2^ Department of Food Science and Technology Faculty of Agriculture Tarbiat Modares University Tehran Iran; ^3^ Department of Chemistry Najafabad Branch Islamic Azad University Najafabad Iran; ^4^ Department of Food Science and Technology Shahreza Branch Islamic Azad University Shahreza Iran

**Keywords:** optimization, quinoa, response surface method, sugar ‐free biscuit

## Abstract

A low‐calorie biscuit formulation containing quinoa flour (cultivars TTKK), isomalt, and maltodextrin was optimized using response surface methodology. Optimized samples were evaluated in terms of total phenolic compounds (TPC), sensory properties, and nutritional value while samples containing only wheat flour (Pishgam var.) and sucrose were used as control. Morphology of isolated starch from quinoa was also investigated. The results showed that with increasing amounts of quinoa, isomalt, and maltodextrin ΔE and Browning index increased, whereas hardness and L values decreased. The formulation containing 25% quinoa flour, 3.5% maltodextrin, and 10% isomalt was found to be optimal with an overall desirability value of 0.95. The sensory evaluation showed that replacement of wheat flour with 25 g/100 g quinoa flour in biscuits was acceptable. TPC of the optimal biscuit (1,180.34 ± 0.02 μg GAE/g) was higher than that of the control sample (729.95 ± 0.007 μg GAE/g). In addition, the optimized biscuit had more protein (8.36 ± 0.035%) and dietary fiber (2.14 ± 0.035%) content compared with the control sample (7.01 ± 0.007% and 1.66 ± 0.028%, respectively). The consumption of 100 g of optimized quinoa biscuits supplies the daily requirement of Fe, Mg, Ca, and Zn at 2.43%, 44.81%, 19.46% and 1.12%, respectively.

## Graphical abstract

A low‐calorie biscuit formulation containing quinoa flour (cultivars TTKK), isomalt, and maltodextrin was optimized using response surface methodology. The results showed that with increasing amounts of quinoa, isomalt, and maltodextrin ΔE and Browning index increased, whereas hardness and L values decreased. The formulation containing 25% quinoa flour, 3.5% maltodextrin, and 10% isomalt was found to be optimal with an overall desirability value of 0.95. The sensory evaluation showed that replacement of wheat flour with 25 g/100 g quinoa flour in biscuits was acceptable. In this study, the low‐calorie biscuit formulation was optimized using quinoa flour (cultivars TTkK), isomalt, and maltodextrin according to Response surface method. Optimized samples were evaluated in terms of total phenolic compounds and nutritional value while samples containing wheat flour (Pishgam var. and sucrose) were used as control.

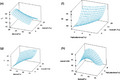

## 1 INTRODUCTION

Biscuit is a popular product consumed worldwide due to its high nutritive value. Biscuits and other sweet bakery products are rich in sugar (mainly sucrose) and fat, thus high in calories (Lee et al., 2020). It is well known that the excessive consumption of sugar increases the energy intake, leading to diseases, such as diabetes and obesity (Milićević et al., 2020). Functional properties of biscuits can be increased by modifying or improving the major ingredients. These modifications can be achieved by replacing sugar and fats by ingredients, such as polyols and maltodextrin (Aggarwal et al., 2016). Also, these products can be enriched easily (Dauda et al., 2018) (Bouazizi et al., 2020).

Sweeteners play an important role in providing color, flavor, appearance, taste, and dimension to the finished product. Use of polyols and other bulk sweeteners as the sucrose substitutes for the development of low‐calorie products has been the subject of intense research. Isomalt is an alcoholic and natural sugar that can serve as a good alternative to sugar and its calorie is half of it. Isomalt is currently used in a wide range of confectionery, chocolate, baked goods, pharmaceutical, and functional products, including hard candies, lollipops, chewing gum, breath mints, cough drops, throat lozenges, chocolates, fudge, cookies, and wafers (McNutt & Sentko, 2003).

Maltodextrin is also a class of carbohydrates extracted from a range of botanical sources. It is industrially produced by the enzymatic or acid hydrolysis and is used in a wide range of foods and beverages(Ghandehari Yazdi et al., 2017). Several researchers have studied the effect of replacing sugar in cookie and biscuits (Zucco et al., 2011) (Aggarwal et al., 2016; Mieszkowska & Marzec, 2016). However, few studies have been conducted on replacing sugar with isomalt and maltodextrin in biscuit. Whole grains increase the nutritional profile of the products because they are a rich source of dietary fiber, antioxidants, minerals, and phenolic compounds, which can protect against obesity, cancer, diabetes, and cardiovascular diseases (Pathak et al., 2016).

Quinoa (*Chenopodium quinoa*) is an endemic crop of the Andean region (Demir & Kilinc, 2017; Stikic et al., 2012). It has been recognized as a very nutritious grain because of its high quality and the quantity of protein and essential fatty acids (omega‐3 and 6), as well as carbohydrates with low glycemic indices (Vega‐Gálvez et al., 2018). The seed of the quinoa plant has been called both a pseudooil and pseudocereal seed because of its nutritional profile (Nowak et al., 2016). According to the reports of Food and Agriculture Organization (FAO) regarding amino acid balance, quinoa is one of the few plants that can provide all amino necessary acids, such as thionic and lysines (Diaz‐Valencia et al., 2018; Jiang et al., 2020; Nowak et al., 2016). They are easy to digest because of having no gluten and are unusually complete foods because they possess a well‐balanced set of essential amino acids for humans. they can also serve as a good source of protein (12–18 g/100 g on dry weight), fiber, vitamins (e.g., C, E, and B complex), and important minerals (e.g., Fe, Ca, K, Mg, P, and Zn) (Diaz‐Valencia et al., 2018). Moreover, quinoa is a great example of "functional foods," which may help to reduce the risk of various diseases (Stikic et al., 2012).

A good number of studies have been done on the use of quinoa flour (QF) in bread (Machado Alencar et al., 2015; Turkut et al., 2016), biscuit and cookies (Brito et al., 2015; Goyat et al., 2018). However, little research has been published on the interaction of QF, isomalt, and maltodextrin on the characteristics of sugar‐free biscuit. Therefore, the aim of this study was to use response surface methodology (RSM), as the optimization technique, to produce sugar‐free quinoa biscuit containing isomalt and maltodextrin as sweeteners and to evaluate the effect of different levels of these ingredients on texture, color, and sensory properties of the biscuit. Also, characteristics of optimized sample were compared with sample containing wheat flour and sugar as the control sample.

## 2 MATERIALS AND METHOD

### 2.1 Materials

Wheat flour (Pishgam wheat variety, Agricultural Research Center, Isfahan, Iran), Quinoa flour (QF) (TTKK Karaj, Iran), bakery shortening (Ladan Co., Iran), isomalt (Cargill Co., Germany), maltodextrin (Foodchem International Corp., China), stevioside (Fooding Co., China), soya lecithin (Behpak Co., Iran), ammonium bicarbonate and sodium bicarbonate (SRL Co., India), spray‐dried skimmed milk powder (Zarinshad Co., Iran), spray‐dried egg powder (Golestan powder Co., Iran), and vanilla flavor (Curt Georgy Co., Germany) were obtained. All chemical materials and reagents used were purchased from Merck Co. (Germany). Quinoa seeds were manually dehulled to remove the pericarp. The dehulled seeds were then soaked in water to extract saponins, and were dried and milled to pass through the sieve with 100‐500µ pore size. The characteristics of quinoa flour are presented in Table 1.

**TABLE 1:** Chemical composition of Quinoa and Wheat flour

	Wheat flour	Quinoa flour
Moisture (%)	12.4 ± 0.07^a^	8.96 ± 0.03^b^
Ash (%)	1.2 ± 0.01^b^	2.8 ± 0.08^a^
Fat (%)	1.8 ± 0.04^b^	3.72 ± 0.14^a^
Protein (%)	10.2 ± 0.08^b^	15.1 ± 0.33^a^
Carbohydrate (%)	70.25 ± 0.012^a^	64.83 ± 0.023^b^
Amylose (%)	19 ± 0.06^a^	9.19 ± 0.83^b^
a*	‐0.66 ± 0.04	‐0.23 ± 0.03
b*	9.93 ± 0.02	7.49 ± 0.04
L*	90.93 ± 0.07	88.79 ± 0.11
475 (μm)	0^a^	0^a^
180 (μm)	14.61^a^	15.02^a^
125 (μm)	46.78^a^	47.31^a^
125 (μm)	36.33^a^	35.19^b^

Note

Results are the average of two trials ± standard deviation.

Conversion factors; N x 5.7for wheat flour; N x 6.25 for quinoa flour.

Values are dry weight basis.

Different letters means that there are significant differences between data (p˂0.05).

### 2.2 Proximate analysis of flour

Chemical composition of quinoa and wheat flour, including moisture content, protein, crude fiber, and ash content, were determined in triplicate, on AACC methods No. 44–16, 46–12, 30–10, and 08–01, respectively (AACC, 2000).

### 2.3 Starch isolation and determination of amylose content in flour

Isolation of starch from quinoa and wheat flours was performed according to (Steffolani et al.,^2015^) method with some modification. The isolated starch was then dried at 30℃ for 24 hr, ground to powder, and then stored in a closed dry container until further analysis. Amylose content was evaluated based on the colorimetric determination of amylose by iodine binding (Morrison & Laignelet, 1983).

### 2.4 Morphology of starch granules

The morphology of the starch samples (TTKK quinoa and Pishgam wheat var.) was characterized by scanning electron microscopy (*SEM*‐Zeiss‐ EVO, Germany). For *SEM* analysis, the starch granules were coated with a thin layer of gold and then analyzed at a voltage of 10kV and 5 KX magnification.

### 2.5 Experimental design

Response surface methodology (RSM) with central composite design (CCD) was applied to evaluate the effect of independent variables as quinoa (X_1_) (10–50 wt.%), isomalt (X_2_) (5–20 wt.%) and maltodextrin (X_3_) (1–7 wt.%) on the dependent variables (banding, hardness, L, a, b, browning index (BI), ΔE and sensory properties) in Minitab software V. 10.0.10. A total of treatments (20 run) for biscuit preparation with six replicates at the center point were obtained to determine pure error and repeatability of all data, according to a central composite design.

Table 2 shows the complete experimental design used for biscuit preparation formulated with quinoa, isomalt, maltodextrin, and actual levels of the independent variables. Data were fitted to a second‐order Equation (1) as a function of dependent variables (y_i_).

(1) $$yi=\beta 0+\sum_{i=1}^{3}\beta ixi+\sum_{i=1}^{2}\sum_{j=2}^{3}\beta ijxixj+\sum_{i=1}^{3}\beta iixi^{2}$$

**TABLE 2:** Experimental results of sugar free biscuit quality for response surface analysis

RUN	X1 QF (%)	X2 isomalt (%)	X3 maltodextrin (%)	Hardness (N)	Bending (N)	Surface per diameter (cm)	a*	L*	b*	*BI*	*ΔE*	Overall acceptability
1	40	8.75	5.5	490 ± 7.07	0.59 ± 0.14	45.99 ± 1.23	5.485 ± 0.07	50.72 ± 0.39	20.33 ± 0.33	58.09 ± 0.38	13.19 ± 0.50	3.33 ± 0.98
2	30	5	4	574.5 ± 82.23	0.66 ± 0.24	47.05 ± 2.24	3.615 ± 0.23	57.22 ± 0.02	20.445 ± 0.10	47.9 ± 0.57	9.23 ± 0.18	3 ± 0.42
3	30	12.5	1	278.75 ± 20.87	0.465 ± 0.04	48.01 ± 2.56	3.16 ± 0.21	56.955 ± 0.70	19.88 ± 0.02	46.08 ± 0.90	9.98 ± 0.25	4.16 ± 0.83
4	30	12.5	4	606.25 ± 19.44	0.8 ± 0.08	44.81 ± 1.15	5.16 ± 0.02	55.59 ± 0.02	20.35 ± 0.49	51.51 ± 1.28	9.72 ± 0.39	4.58 ± 0.79
5	30	12.5	4	603.75 ± 24.39	0.815 ± 0.03	45.62 ± 0.89	5.16 ± 0.04	55.585 ± 0.04	20.84 ± 0.22	52.86 ± 0.50	9.34 ± 0.19	4.50 ± 0.79
6	20	16.25	2.5	746 ± 90.12	0.445 ± 0.16	44.02 ± 1.98	5.3 ± 0.48	56.955 ± 0.94	22.625 ± 0.02	56.36 ± 1.74	7.13 ± 0.47	3.58 ± 0.79
7	30	12.5	7	776.5 ± 9.19	0.415 ± 0.007	44.19 ± 2.10	6.305 ± 0.28	52.045 ± 0.31	21.25 ± 0.12	60.28 ± 0.48	11.5 ± 0.31	3.41 ± 0.66
8	40	16.25	5.5	895 ± 62.22	0.75 ± 0.08	46.84 ± 2.21	5.735 ± 0.03	51.59 ± 1.30	19.87 ± 0.14	55.85 ± 1.35	12.75 ± 1.08	2.91 ± 0.99
9	40	8.75	2.5	331.5 ± 64.34	0.485 ± 0.12	51.06 ± 1.76	4.075 ± 0.04	53.99 ± 0.45	18.02 ± 0.21	45.38 ± 0.04	12.71 ± 0.41	2.75 ± 1.05
10	10	12.5	4	909.5 ± 7.77	0.6 ± 0.21	45.55 ± 2.34	3.75 ± 0.09	61.225 ± 0.65	24.405 ± 0.09	54.11 ± 1.09	4.85 ± 0.10	3.7 ± 0.75
11	30	12.5	4	596.25 ± 22.27	0.825 ± 0.09	45.66 ± 0.99	5.175 ± 0.03	55.57 ± 0.04	20.7 ± 0.01	52.51 ± 0.03	9.46 ± 0.04	4.58 ± 0.79
12	30	12.5	4	601.5 ± 36.06	0.785 ± 0.03	43.63 ± 1.09	5.16 ± 0.05	55.565 ± 0.03	20.785 ± 0.16	52.73 ± 0.41	9.4 ± 0.08	4.58 ± 0.79
13	20	8. 75	2.5	432.5 ± 82.73	0.45 ± 0.05	44.62 ± 1.0	4.89 ± 0.16	57.815 ± 0.33	23.405 ± 0.02	56.91 ± 0.69	6.16 ± 0.16	3.70 ± 0.86
14	20	16.25	5.5	968 ± 45.96	0.5 ± 0.0	48.18 ± 1.70	6.93 ± 0.41	53.955 ± 0.57	23.605 ± 0.19	65.63 ± 0.86	8.59 ± 0.58	3.66 ± 0.88
15	20	8.75	5.5	780 ± 73.18	0.67 ± 0.05	45.13 ± 0.90	6.075 ± 0.10	56.7 ± 0.60	23.92 ± 0.07	61.41 ± 0.89	6.24 ± 0.43	3.29 ± 0.45
16	30	12.5	4	598.25 ± 27.22	0.79 ± 0.042	49.83 ± 1.87	5.15 ± 0.04	55.59 ± 0.04	20.845 ± 0.21	52.85 ± 0.71	9.34 ± 0.14	4.58 ± 0.79
17	40	12.5	4	524.5 ± 38.89	0.535 ± 0.14	33.36 ± 2.10	4.62 ± 0.15	52.855 ± 0.14	18.425 ± 0.27	48.47 ± 0.83	12.99 ± 0.32	2.75 ± 1.05
18	40	16.25	2.5	818.75 ± 78.85	0.51 ± 0.18	47.11 ± 20	5.58 ± 0.014	52.015 ± 0.09	20.17 ± 0.05	55.96 ± 0.06	12.25 ± 0.10	3.25 ± 0.96
19	30	12.5	4	601 ± 38.18	0.78 ± 0.05	45.55 ± 1.70	5.17 ± 0.04	55.595 ± 0.03	20.835 ± 0.20	52.85 ± 0.66	9.34 ± 0.14	4.58 ± 0.79
20	30	20	4	747.25 ± 74.69	0.49 ± 0.29	52.60 ± 1.60	8.78 ± 0.18	45.935 ± 0.7	20.62 ± 0.29	72.26 ± 0.77	17.16 ± 0.86	3.66 ± 1.23

Where, *β_0_, β_i_, β_ij_* are the regression coefficients and *x_ij_* is the independent parameter and *y_i_* is the response. The adequacy and fitting quality of equations assessed for dependent variables were tested using analysis of variance (ANOVA) at a significance level of 0.05.

### 2.6 Optimization of parameters

Optimization of multiple responses was carried out by using numerical optimization technique of the Minitab software, version 16. The quinoa flour, maltodextrin, and isomalt were kept in range while hardness, ΔE were minimized and overall acceptability was targeted to be similar to the control sample. Finally, the optimal sample was compared with the control sample in terms of texture, sensory properties, protein, and nutritional value.

### 2.7 Biscuit preparation

Biscuits were prepared in the Kamvar Company (Producer of various sugar‐free and diet products). Control biscuits were prepared using the creaming method adopted by Raju et al. (2007) with slight modifications. Bakery shortening (24.4 wt %) was creamed using Hobart mixer at a high speed (240 rpm) until its volume was doubled. Isomalt and maltodextrin were weight according to design experiment treatment (Table 2) and mixed with the foamed cream along with lecithin (1.66% wt), sorbitol (1.65% wt), skimmed milk powder (2.66% wt), spray‐dried egg powder (1.32% wt), sodium bicarbonate (0.6% wt), ammonium bicarbonate (0.1% wt), stevioside (0.02%), vanilla aroma (0.2% wt), and water (10% wt) for 10 min at a low speed (55 rpm) (all percentages are based on the flour weight). Then, the flour was added and mixed for 2 min at 55 rpm. According to experimental design of treatments (Table 2), biscuits were made by replacement of wheat flour with quinoa flour for treatment and control sample formulated with wheat flour and sucrose as sweetener. The dough was fed into the forming machine and biscuits with the thickness of 5 mm were collected on a baking tray and baked at 210℃ for 20 min, this was followed by cooling at room temperature for 20 min. The biscuits were packed in low density polyethylene (LDPE) bags (0.2 mm thick) and stored at 25℃ for further analysis.

### 2.8 Physical characteristics of biscuits

The thickness (T) and diameter (D) of biscuits were measured according to AACC methods to calculate spread ratio of samples (AACC??). This response was measured as the ratio of diameter to thickness (Demir & Kilinc, 2017). Hardness and bending analysis of the biscuit samples were determined via the three‐point bend test which was performed using a TAXT2 texture analyzer equipped with the three‐point bending rig (HDP/3 PB), according to the method described by Brito et al. (2015).

Texture analyzer settings were the pretest speed of 0.5 mm/s, the test speed of 3.0 mm/s, and the posttest speed of 10.0 mm/s, at a distance of 5 mm. The applied load cell was 50 kg. The maximum force at break (N) and the mean distance at break (mm) were recorded. The color parameters were measured using HunterLab ColorFlex (Reston, VA). Averages of three parameters of L* (brightness; 0: black, 100: white), a* (+a: redness; ‐a: greenness), and b* (+b: yellowness; ‐b: blueness) values for flour and biscuits were recorded (Tables 1 and 2). Moreover, *BI* (browning Index) and *∆E* (total color difference) of the biscuits were measured according to the Equations(2, 3, and 4) (Pourabedin et al., 2017).

(2) $$BI=\frac{\left[100\times \left(x‐0.31\right)\right]}{0.17}$$

(3) $$\mathrm{Where}\;X=\frac{\left(a*+1.75\times L*\right)}{\left(5.645\times L*+a*‐3.012\times b*\right)}$$

(4) $$\Delta E=\sqrt{\left(*\Delta a\right)2+\left(*\Delta b\right)2+\left(*\Delta L\right)2}$$

Where Δa, Δb, and ΔL are difference between color parameter for standard and sample.

### 2.9 Sensory evaluation

To ensure the ethical acceptability of the experiments, we got the Human Ethics Research Committee certificate from Research Ethics Committees of Islamic Azad University on October 29, 2020 with the approval ID of IR.IAU.NAJAFABAD.REC.1400.062. All biscuit samples were evaluated in terms of texture and overall acceptability on a five‐point hedonic scale scoring from one (lowest) to five (highest), using a panel of 20 trained panelists. Mean scores given by the panelists were used for the statistical analysis. Sensory evaluation of the optimized biscuit sample was also carried out by those 20 panelists. Sensory evaluation of the obtained biscuits was done in accordance with the methods previously used by Pourabedin et al., (2017).

### 2.10 Proximate composition and chemical analysis of optimized biscuits

Chemical analysis was performed on control and optimized samples. Biscuit samples were ground with a laboratory mill (Panasonic MX ‐J120‐P, Japan) until a fine powder obtained. Moisture, crude fat, crude fiber, carbohydrate, and protein content were measured according to the AACC methods described for flour analysis. Measurements were made in triplicate.

The carbohydrate content was calculated by difference. Total carbohydrates were measured according to the following equation:

(5) $$\left[\text{Carbohydrate}\;\left(\%\right)\;=100\;‐\;\left(\text{Moisture}\%\;+\;\text{Fat}\%+\;\text{Protein}\%\;+\;\text{fiber}\%\;+\;\text{Ash}\%\right)\right]$$

Moreover, energy was calculated by the Atwater method (Osborne and Voogt 1978). The energy value was calculated according to the Atwater equation (FAO/WHO/UNO, 1994). Water activity (aw) of the biscuits was measured by Lab Master‐aw (Switzerland) at 25 ˚C. Determination of total phenolic compounds was performed according to Pourabedin et al. (Pourabedin et al., 2017) with some modification. One gram of biscuit powder was mixed with 10 ml acidified methanol/HCl 1% (v/v) for 24h at ambient temperature. After that, the mixture was centrifuged at 5,000 g for 20 min. The obtained supernatant was used for phenolic compounds measurement by folin‐ciocalteu reagent at 765 nm. Gallic acid was used as standard and total phenolic compounds were declared as gallic acid equivalents (mg GAE/G dry weight).

### 2.11 Nutritional properties

The minerals of samples (Ca, Mg, Fe and Zn) were determined by atomic absorption spectrophotometry (Perkin Elmer Model 3,300) according to AOAC methods (AOAC, 2006). The results were obtained in triplicate and expressed in g/100 g. Dietary Fiber of control and optimized biscuit samples was measured according to AACC (2000) standard method No. 32–07.01.

### 2.12 Statistical analyses

Minitab software, version 16, was used to evaluate the effect of independent variables on multiple responses. Analysis of data generated during the present investigation was carried out using RSM by employing CCD to generate the combination of factors leading to the better quality of the biscuit (Table 2). The optimize response was determined through designing experiments, fitting the mathematical models and finally, selecting the levels of variables. Analysis of variance (ANOVA) was carried out for each response at significance level of.05. Statistical software SPSS V.17 was used for LSD test to compare means of the results.

## 3 RESULTS AND DISCUSSION

### 3.1 Proximate composition of flours

The chemical composition of the quinoa and wheat flours is shown in Table 2. The amount of protein, fat, ash, and crude fiber in the quinoa flour were significantly (p ˂ .05) higher than those of Pishgam wheat. Some varieties of quinoa have been reported to have protein content in the range of 12.02%–19.59% (Contreras‐Jimenez et al., 2019; Steffolani et al., 2015). Total carbohydrate content of quinoa flour (64.83 ± 0.023 ) was observed to be within the values of (66.63%–72.84%) (Valdez‐Arana et al., 2020). Results showed that the amount of crude fiber in quinoa was 6.3 ± 0.05 which was higher than that of Pishgam wheat, it was within the range of 1.92%–9.48% in agreement with those reported by Valdez et al., Nowak et al. (Nowak et al., 2016; Valdez‐Arana et al., 2020). Based on the above results, TTKK quinoa has good nutritional value and can be used as a nutritional food source in confectionery products.

### 3.2 Characteristics of starch

**FIGURE 1:**
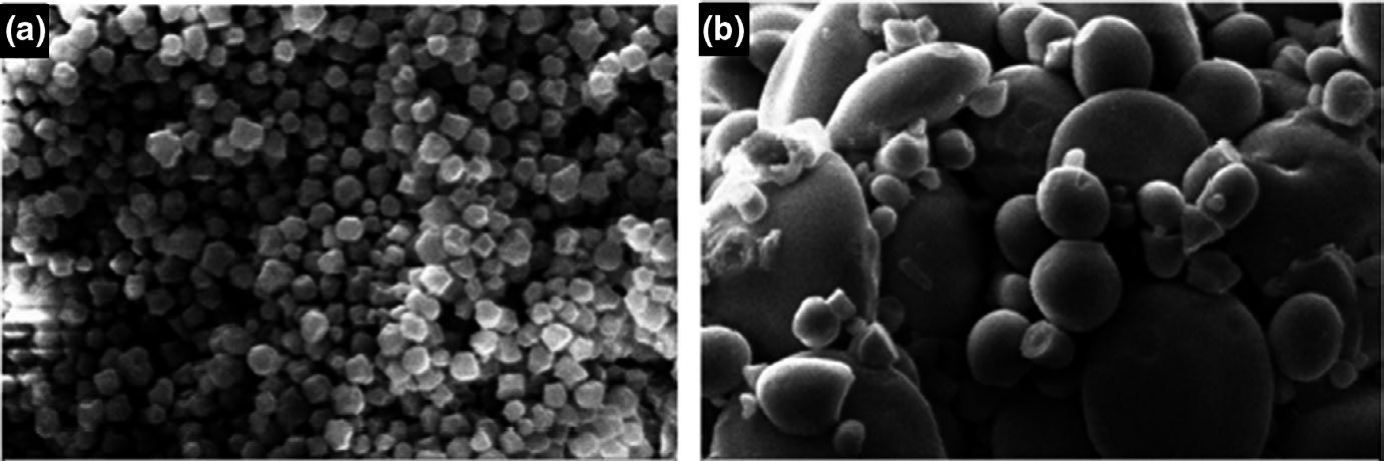
Scanning electron micrographs of starch granules from (a) TTKK quinoa flour and (b) Pishgam flour. (5.00 K× magnification)

The amylose content of quinoa starch was 9.19% ± 0.83 which was significantly lower than the amylose contents of the wheat (19.57% ± 0.66), however, amylose content of some quinoa varieties reported by Jiant et al. (2020) (QS, MS, and PS) was higher than that of measured in this study. According to Steffolani (2013), the amylose content of three varieties of quinoa (Q Jacha Grano/ Q Kurmi/Q Chucapaca) was in the range of 8.22%–9.3%. Generally, quinoa starch with low amylose content (amylose less than 15% ) is classified as waxy starch and thus cannot be easily retrograded (Bertolini, 2009). The morphological characteristics of the quinoa and wheat starches were also evaluated. Scanning electron micrographs of the starch granules of the both flours demonstrated that shape and size of starch granules had significant differences. Isolated quinoa starch had spherical and polygonal shape with submicron size (Figure 1a). In addition, granule surfaces of quinoa were less smooth than those of wheat and potato starch granules. This morphological characteristics was the same as those reported previously (Fuentes et al., 2019; Jan et al., 2017).

Regarding the morphology and size of the wheat starch, the *SEM* indicated a bimodal pattern in which large starch granules were surrounded by many small starch granules (Figure 1b). Pishgam wheat starch showed a granular size distribution between 2–10 µm whereas quinoa starch granules had much smaller sizes (0.5–1 µm). It is noteworthy that the size of TTKK quinoa starch granules was significantly smaller than those of PS and MS quinoa varieties (44.65 and 14.20 μm, respectively) which was reported by Jiang et al. (2020) and Jan et al., 2017; Lindeboom et al., 2005, who reported average diameter of starch granules of quinoa species to be in the range of 0.4–3.5 μm. There are reports that quinoa granules are eligible to form aggregate structures. The starch granules of the TTKK quinoa can be classified as the microgranular starch (˂5 µm) which could play an important role in producing formulations with creamy mouthfeel while containing low fat/sugar (Lindeboom et al., 2004, 2005).

### 3.3 Model fitting by response surface methodology

Response surface methodology (RSM) with central composite design (CCD) was used to investigate the effect of the independent variables on the dependent variables. The experimental data obtained by the response variables are shown in Table 1. For evaluation of the model's adequacy, different parameters including the model *F*‐value, the coefficient of determination (*R^2^*), *F*‐value (lack of fit) and coefficient of variation (CV) were considered according to ANOVA technique (Table 3). In order to predict the effects of variables on the responses and to expand the response surface models, all insignificant terms (with p > .05) were eliminated (Table 3) and the final regression equations were developed as in equations 6–11:

**TABLE 3:** Analysis of variance (ANOVA) for determination of model fitting, Regression coefficient (β), coefficient of determination (Adj. *R* ^2^) and *F*‐test value of the predicted second order polynomial models for the responses

Factor	Coefficient (β)						
	Hardness	bending	L*	ΔE	BI	Overall acceptability	Surface per diameter
Intercept	602.381	‐0.543	62.01	2.524	63.867	‐3.866	45.29
X1: quinoa (%)	‐31.471*	0.019	‐0.48^**^	0.543^**^	‐0.308	*0.19*	*1.15*
X_2_ : Isomalt (% )	‐25.535^**^	0.061	1.17**	‐0.893^**^	‐2.82**	*0.441*	*‐2.17*
X3: maltodextrin (%)	208.681^**^	+0.314	0.0836*	‐0.508	2.256*	*2.685*	‐1.22
x_1_x_2_	0.921	‐0.16	0.0058	‐0.01	0.011	*0.014*	*‐0.013*
x_1_x3	‐1.974	‐0.20	0.0024	‐2.48	‐0.0069	‐0.025	0.0589
x_2_x_3_	‐3.268	+0.38	0.015	‐0.0033	‐0.126	‐0.027	0.132
X_1_^2^	0.357	‐5.21^**^	0.0035	0.022	‐0.0023	‐0.0049^*^	‐0.0137*
X_2_^2^	1.54	‐0.0035^**^	‐0.072**	0.054^*^	0.154*	‐0.032*	0.869
X_3_^2^	‐5.15	‐0.037^**^	‐0.125	+0.0677	0.199	‐0.021^*^	0.13
Adj. *R* ^2^ (%)	88.02	84.38	85.20	88.1	78.55	74.5	76.89
*F*‐value(model )	6.81**	6.14^**^	6.4^*^	8.09^**^	4.07^*^	3.14^*^	1.45
*F*‐value(lack of fit)	2.01	0.47	4.17	1.53	1.21	0.53	4.38

^*^Significance level: *p* *≤* 0.05; ^**^Significant level: *p ≤* 0.01.

Final equation:

(6) $$\text{Hardness as}\;\left(y\right)=602.381-31.471\;{\text{x}}_{1}+25.535\;{\text{x}}_{2}+208.681\;{\text{x}}_{3}$$

(7) $$\text{Bending value as}\;\left(y\right)=-0.5431-5.2100\;{\text{x}}_{1}^{2}-0.00357\;{\text{x}}_{2}^{2}-0.0373\;{\text{x}}_{3}^{2}$$

(8) $$\text{L value as}\;\left(y\right)=62.0107-0.4868{\text{x}}_{1}+1.174{\text{x}}_{2}+0.0836{\text{x}}_{3}-0.072{\text{x}}_{2}^{2}$$

(9) $$\text{BI as}\;\left(y\right)=63.867-2.820{\text{x}}_{2}+2.256{\text{x}}_{3}+0.154{\text{x}}_{2}^{2}$$

(10) $$\Delta \text{E as}\;\left(y\right)=2.5240+0.543{\text{x}}_{1}-0.8936{\text{x}}_{2}+0.054{\text{x}}_{2}^{2}$$

(11) $$\text{Spread ratio as}\;\left(y\right)=45.29-0.0137{\text{x}}_{1}^{2}$$

(12) $$\text{Overall acceptability as}\left(y\right)=-3.886-0.0049{\text{x}}_{1}^{2}-0.032{\text{x}}_{2}^{2}-0.212{\text{x}}_{3}^{2}$$

### 3.4 Physical characteristics of biscuits

#### 3.4.1 Biscuit hardness

**FIGURE 2:**
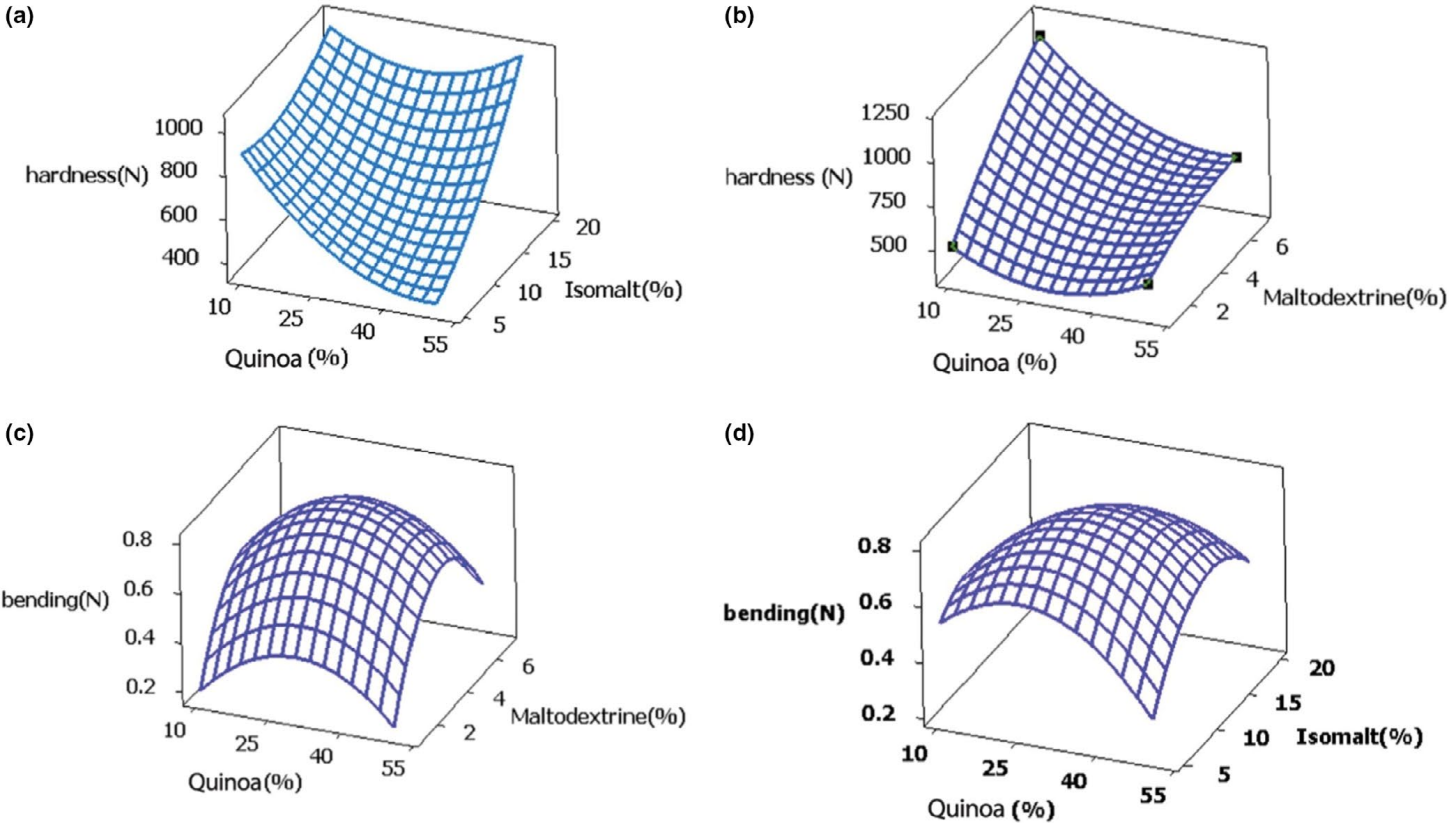
3d response surface plot of (a) quinoa, isomalt on hardness; (b) quinoa and maltodextrin on hardness; (c) quinoa and maltodextrin on bending test; (d) quinoa, isomalt on bending test

The hardness measured for different treatments is presented in Table 2. Results showed that hardness values ranged from 331.5 ± 64.34 to 968 ± 45.96 N. According to the results, maximum hardness was observed in run = 14 with 20% quinoa, 5.5% maltodextrin, and 16.25% isomalt. The coefficient of determination (R^2^) was 88.02 and the proposed model for hardness was defined in accordance with Equation 6, regarding the coefficients obtained. This model showed that maltodextrin and isomalt had a positive linear effect, whereas QF had a negative linear effect (Table 3 and Eq:6) on hardness. As can be seen in Figure 2a and b, the hardness value of the biscuit significantly decreased with increasing levels of QF. This was probably due to the high amount of fat, fiber, and lack of gluten in QF. Since less interaction between starch and protein could lead to the reduction of hardness in the biscuit. Fat, acting as a lubricating agent, could make a soft texture and reduce the hardness value of the biscuits. In addition, lack of gluten in QF could prevent the formation of the elastic network (Goyat et al., 2018). Demir and Kilinç have reported that partial replacement of WF with QF has more advantages in the production of cookies from the weak wheat, as it could improve the texture of the cookies (Demir & Kilinc, 2017).

To explain the results obtained in this study, the presence of insoluble fibers in the QF could lead to the collapse of the gluten network, thus reduce the hardness. These results, however, contradicted those of Brito et al. (2015). They reported that the decrease in QF led to the increased of the hardness value in a cookie (Brito et al., 2015). It could be due to different variety of WF and QF and the composition of the cookies. On the other hand, the hardness value enhanced significantly with the increase of maltodextrin due to the maltodextrin gelation/gelatinization (increasing viscosity) in the presence of water; this could immobilize a part of water and markedly reduce the water available for gluten to hydrate and enhance the hardness (Savitha et al., 2008). Also, the formation of gels was the result of the interactions between the components of the spiral amylose and the amylopectin molecule; as it is evidenced in the texture strength of NaanBerenji (an Iranian confectionary) (Ghandehari Yazdi et al., 2017). The increment of isomalt up to 12% enhanced the biscuit hardness rapidly. It was similar to what was found in a study by Pareyt and co‐authors (Pareyt et al., 2011). Regarding the effect of variables on hardness, it can be concluded that low levels of QF and the high levels of isomalt and maltodextrin led to the production of a biscuit with higher hardness, probably due to the interaction of QF, isomalt, and maltodextrin.

One reason for hardness decrease with increasing quinoa level is the morphology of starch and the difference in the amount of amylose and amylopectin of quinoa and wheat. According to results of this study, shape and size of quinoa and wheat starch granules are significantly different. Isolated TTKK quinoa starch has a spherical and polygonal shape with submicron size (Figure 1a). Quinoa starch granule surface was less smooth than that of wheat. The shape of granules and size of starch with amylose/amylopectin ratio influence the physicochemical and functional properties of starches and ultimately affect product characteristics (Valdez‐Arana et al., 2020).

Bending value shows the flexibility of biscuits. Biscuit bending value was in the range of 0.415 ± 0.01 to 0.825 ± 0.09 *N* (Table 2). As shown in Table 3, the quadratic effects of variables were significant (p < .05). Moreover, results of ANOVA revealed that the interaction of variables, QF ×Isomalt, QF×maltodextrin and isomalt ×maltodextrin, had no significant effect on the flexural modulus, and proposed model for this response indicated in Eq.7. The coefficient of determination (R^2^) for SWF was 84.35% (Table 3). According to 3D response plots in Figure 2c and d, the maximum bending force value was observed in the middle level of variables. The bending force decreased significantly due to the decrease in the moisture content of the biscuits with increasing the amount of QF from 25% to 50% in the formulation. It also could be attributed to the high amounts of fat, protein (high water absorption), and insoluble fiber in quinoa flour, which reduced the flexibility of the biscuits as QF level increased in the formulation. This was probably due to the formation of new bonds and the interactions between gluten proteins and proteins at the surface of QF (Stikic et al., 2012). The results of this study showed that by increasing the amount of QF, maltodextrin, and isomalt in the dough formulation, a compact texture was created, the fractional modulus decreased and the hardness of the biscuit increased. It corresponded to Bilgicli and Ibanoglu results (Bilgiçli & İbanoğlu, 2015).

### 3.5 Color analysis

Color measurement test results are presented in Table 2. The L* of the samples ranged between 61.22 ± 0.65 and 45.93 ± 0.7. Among the treatments, the samples with 30% quinoa, 4% maltodextrin, and 20% isomalt (run = 20) had a darker color than the other samples. ANOVA results (Table 3) showed that linear effect of all three variables and quadratic effect of isomalt were significant (p < .05). Moreover, comparison of *F*‐value variables showed that QF with *F*‐value = 20.32 had a greater effect on L*. Based on the coefficient obtained, the proposed model for this response is as Eq. 8.

**FIGURE 3:**
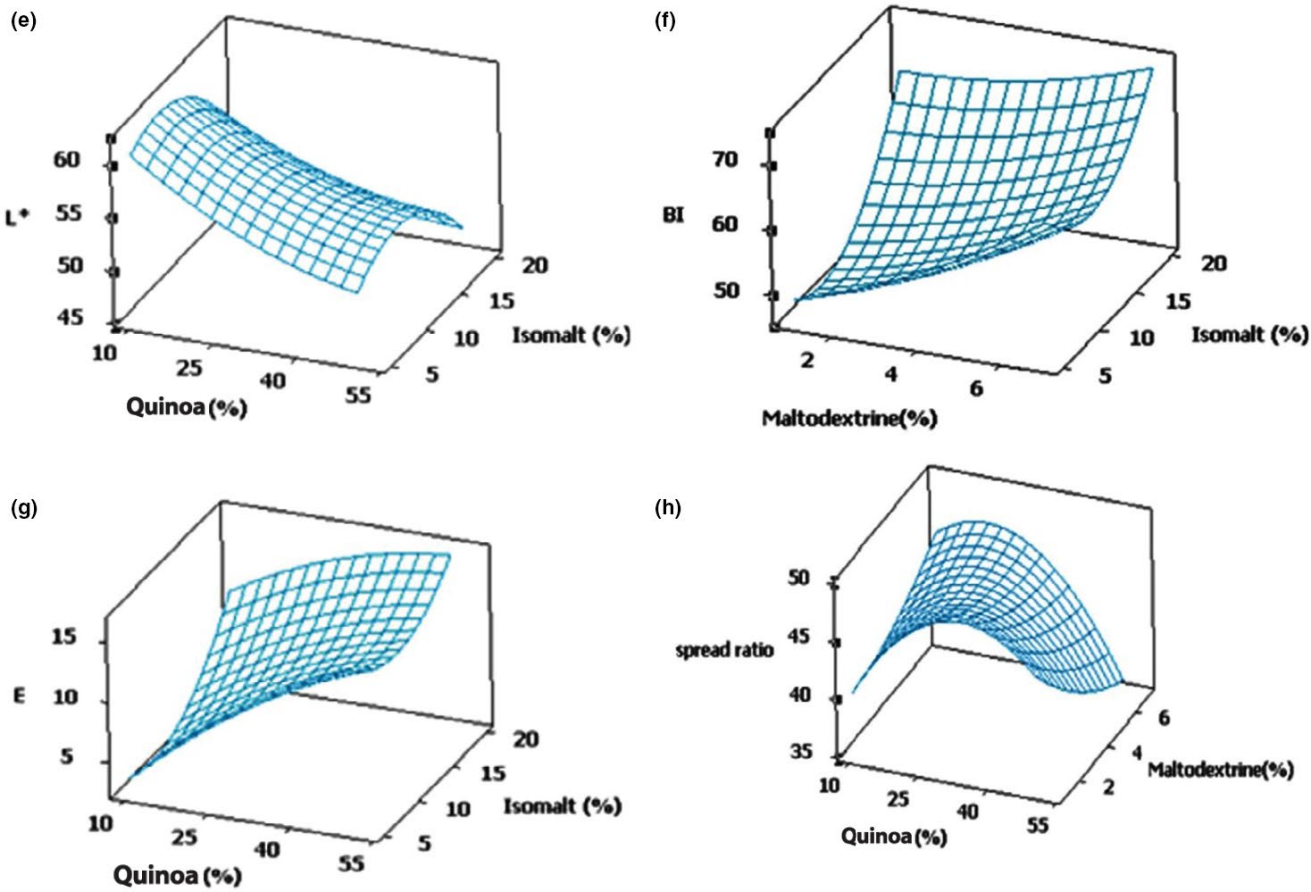
3d response surface plot of (e) quinoa and isomalt on L*; (f) maltodextrin and isomalt on browning index (BI); (g) quinoa and isomalt on ΔE; and (h) quinoa and maltodextrin on spread ratio

The negative coefficients obtained for quinoa showed that increment of quinoa level in the dough reduces brightness of biscuit Figure 3e. Another evaluated response was browning index (BI) which is an indication of browning reaction due to the effects of caramelization and Maillard browning reactions in cereal products (Tamanna & Mahmood, 2015). According to Table 2, “BI” values ranged from 45.38 ± 0.04 to 72.26 ± 0.77.

The ANOVA assay and the coefficient estimates of BI (Eq. 9) showed that the linear effect of isomalt and maltodextrin were significant (p < .05) (Table 3). Evaluation of the results revealed that the color of the biscuits, especially the BI, was affected by the quadratic effect of isomalt (Figure 3f). In addition, linear effect of QF and isomalt was significant on the *∆E* value (p < .01). Among variables, the quadratic effect of isomalt on this response was significant (p < .05). Surface plots (Figure 3g) revealed that increment of QF and isomalt increased *∆E* and BI values of the biscuits (Figure 2f and g). According to Table 1, quinoa flour had lower lightness (L*) in comparison with wheat flour. This was probably due to the presence of the natural dark‐colored pigments, the high protein content and sugars in quinoa seeds. A relationship between lightness and particle size of the flour used in the cookie formulation has already been described (Zucco et al., 2011). However, in this study, the particle size of QF and WF were approximately the same (Table 1). Therefore, particle size had no effect on lightness.

These results were consistent with Demir and Kilinc results. They reported that color of cookie samples became darker when higher level of QF was added (Demir & Kilinc, 2017). It seems that in higher proportions of QF and isomalt, due to the increase of protein, sugar, and phenolic compounds content in the biscuit formula, the rate of Maillard reaction is intensified, and consequently formation of melanoidin pigments increases, resulting in a darker color product. In addition, the formation of dark pigments due to oxidation of phenolic compounds in quinoa flour could be another reason for these results (Adelakun et al., 2012).

When isomalt and maltodextrin were added above intermediate level (12.5% and 4% respectively), the lightness value decreased and the a* value increased. Probably, the high amounts of maltodextrin which increased the reducing sugars could intensify Maillard and caramelization reactions. Isomalt does not have free carbonyl groups to participate in the browning reaction. Nourmohammadi and co‐authors found that alcoholic sugars such as maltitol and xylitol do not have free carbonyl groups to participate in the browning reaction; however, color change in the crust of cake was observed due to heating up to the boiling point and subjecting to thermal decomposition (Nourmohammadi et al., 2011). On the other hand, with the increase of isomalt (above 12.5%) and maltodextrin at the alkaline pH, the BI* value increased because of the increase in the amount of the reducing sugar and the presence of sugars, which promote of the Maillard reaction speed.

### 3.6 Spread ratio

Results showed that spread ratio response was between 33.35 and 52.06 and the minimum amount of this response was seen in run = 17 with 50%, 12.5%, and 5% content of quinoa, isomalt, and maltodextrin, respectively (Table 2). In terms of spread ratio, quadratic effect of quinoa was significant (p <.05). Based on the ANOVA assay, proposed model was introduced in Eq. 11, but F‐ value and p‐value of model were equal to 1.45 and 0.285, respectively. According to the results, the model was not significant (p < .05). Figure 3h indicates the 3D response surface plot of the effect of quinoa and maltodextrin on spread ratio. Maximum values of this response occurred in the middle level of quinoa and maltodextrin. This might be a result of the decrease in the viscosity of the biscuit dough due to the addition of maltodextrin (Savitha et al., 2008). Considering the protein and fiber present in quinoa flour, its influence on the spread ratio of the quinoa biscuit samples could be described.

### 3.7 Optimization of variables and desirability

In response surface method, the desirability function is widely used to determine a combination of variables to optimize multiple responses and provide the most desirable responses. To obtain maximum desirability, the BI was defined as minimum, L* and overall acceptability were set in maximum level and other responses were set in range. The formulation consisting of 25% QF, 3.5% maltodextrin, and 10% isomalt was found to be optimal with an overall desirability value of 0.95. The optimal sample formulation and its components are presented in Table 4.

**TABLE 4:** Formulation of optimized biscuit sample

Ingridient	Optimum sample
Wheat flour wt %	75^a^
Quinoa flour (Qf) wt %	25
Isomalt wt %	10
maltodextrin wt %	3.5
Bakery shortening wt %	24.4
lecithin wt %	1.66
Sorbitol wt %	1.65
Skimmed milk powder wt %	2.66
Spray dried egg powder wt %	1.32
Sodium bicarbonate wt %	0.6
Ammonium bicarbonate wt %	0.1
Stevioside wt %	0.02
Vanilla aroma wt %	0.2

^a^The ingredients are based on 100 units of flour (wheat flour+ quinoa flour).

### 3.8 Sensory analysis

**FIGURE 4:**
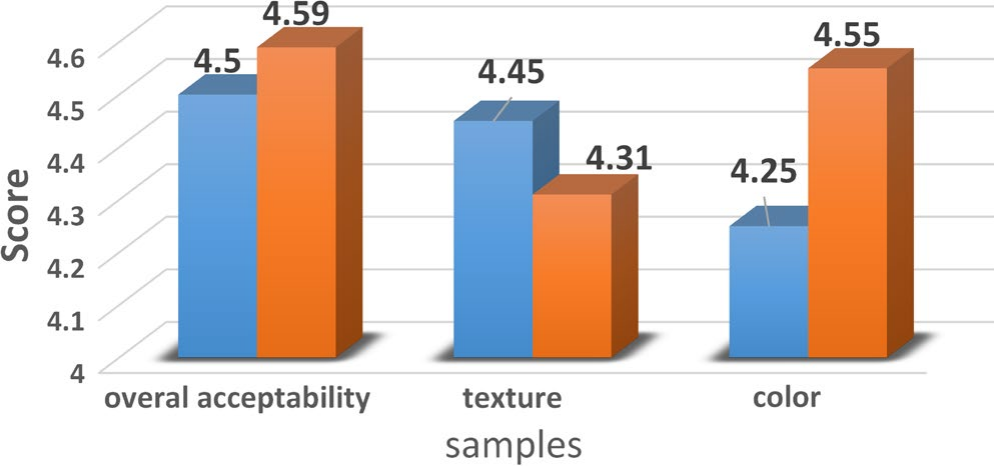
Sensory evaluation of optimized and control biscuits. (control 1: biscuit with 100% wheat flour and sucrose)

The overall acceptability score ranged from 2.75 ± 1.05 to 4.58 ± 0.79 (Table 2). According to the results presented in Table 3, quadratic effect of variables was significant (p < .05). Moreover, negative coefficient of *x_1_^2^, x_2_^2^ and x_3_^2^* in Eq. 12 revealed that addition of isomalt, QF, and maltodextrin in biscuit formulation had adverse effect on the overall acceptability. Also, the overall acceptability increased at an intermediate level of QF, maltodextrin, and isomalt. Figure 4 shows the average score obtained for color, texture, and overall acceptability of optimized and control samples. Control sample was formulated with wheat flour and sucrose as sweetener, while other ingredients were the same as treatments.

### 3.9 Proximate composition and chemical analysis of optimized biscuits

Proximate composition of 100 g of optimized and control biscuit samples are presented in Table 5. Results showed that the optimized biscuit had more protein (8.36 ± 0.035%) and dietary fiber (2.14 ± 0.035%) compared with the control sample with 7.01 ± 0.007% and1.66 ± 0.028%, protein and fiber respectively.

**TABLE 5:** Effect of quinoa flour on the chemical and nutritional properties of biscuit (mean values ± standard deviation)

Composition	Control 1	Control 2	Optimum sample
Fat (%)	17.32 ± 0.007^a^	17.31 ± 0.014^a^	18.02 ± 0.028^b^
Protein (%)	7.01 ± 0.007^a^	7.02 ± 0.021^a^	8.36 ± 0.035^b^
Sugar (%)	18.09 ± 0.035^c^	3.2 ± 0.28^b^	2.90 ± 0.021^a^
Carbohydrate (%)	69.96 ± 0.021^b^	69.86 ± 0.014^b^	65.96 ± 0.042^a^
Energy (kcal/100 g)	463.44 ± 0.028^c^	443.08 ± 0.035^b^	432.68 ± 0.049^a^
Ash (%)	1.08 ± 0.035^b^	1.05 ± 0.021^a^	1.83 ± 0.014^c^
Dietary fiber (%)	1.66 ± 0.028^a^	1.68 ± 0.014^a^	2.85 ± 0.035^b^
Moisture (%)	2.97 ± 0.042^a^	3.08 ± 0.021^a^	2.98 ± 0.028^a^
a_w_	0.34 ± 0.028^b^	0.33 ± 0.014^b^	0.30 ± 0.007^a^
Total phenolic content (µg GAE/g)	729.95 ± 0.007^a^	730.05 ± 0.021^a^	1180.34 ± 0.02^b^
Ca (mg/100g)	15.85 ± 0.070^a^	15.8 ± 0.14^a^	19.46 ± 0.042^b^
Mg (mg/100g)	21.25 ± 0.028^a^	21.31 ± 0.021^a^	44.81 ± 0.028^b^
Fe (mg/100g)	1.52 ± 0.007^a^	1.53 ± 0.028^a^	2.43 ± 0.014^b^
Zn (mg/100g)	0.53 ± 0.021^a^	0.54 ± 0.028^a^	1.12 ± 0.019^b^

Note

Control 1: biscuit with 100% wheat flour and sucrose and Control 2: sugar free biscuit with 100 % wheat flour. Different letters means that there are significant differences between data (*p* ˂ 0.05).

The moisture content and a_w_ level of optimized and control samples indicated that the product has good storage stability. The a_w_ of quinoa biscuits were lower than that of the control sample. In quinoa samples, presence of maltodextrin and isomalt could increase the osmotic pressure in the system, leading to decreased water activity. Another reason for these results could be the high amount of dietary fiber in quinoa flour and quinoa biscuit which affects the water absorption and decline a_w_. This result was similar to that reported in Lowe and Kershaw study (Lowe & Kershaw, 1995).

Recommended dietary allowance (RDAs) for children (4–8 years) is 800 mg of Ca, 10 mg of Fe, 5 mg of Zn, 130 mg of Mg (Demir & Kilinc, 2017). According to Table 5, consumption of 100 g of optimized quinoa biscuit supplies the daily requirement of Fe, Mg, Ca, and Zn at 2.43%, 44.81%, 19.46% and 1.12%, respectively. Moreover, results showed sugar decreased significantly (p < .05) in fortified biscuits. It was due to the replacement of sucrose with isomalt and maltodextrin. In optimized biscuit, replacement of wheat flour by quinoa flour caused a 0.3% decrease of sugar due to the high fiber content of quinoa flour. Therefore, the carbohydrate and energy content of quinoa biscuit samples were significantly reduced (p < .05) (Table 5).

Measurement of total phenolic compounds (TPC) indicated that TPC of the optimal biscuit was 1,180.34 ± 0.02 μg GAE/g which was significantly higher than that of the control sample value (729.95 ± 0.007 μg GAE/g (p < .05) (Table 4). Previous studies have indicated that quinoa seed is a good source of phenolic compounds and bioactive component, such as flavonoids and saponins (Diaz‐Valencia et al., 2018; Vega‐Gálvez et al., 2018).

### 3.10 Conclusions

According to findings of this study, it is suggested that quinoa could be used as a pseudocereal substitute for wheat, and based on the optimization, the formulation consisting of 25% quinoa flour, 3.5% maltodextrin, and 10% isomalt was found to be the optimal composite. Results showed that low sugar biscuits made from quinoa, isomalt, and maltodextrin were good sources of protein, minerals, fiber, and phenolic compounds. The biscuits had decreasing sugar content and calorie with quinoa substitution. The optimized biscuit showed a desirable overall acceptability. These results approved the potential of quinoa biscuit as a functional biscuit which can be considered by diabetics.

## CONFLICT OF INTEREST

The authors declare no conflict of interest.

## AUTHOR CONTRIBUTIONS

**Narges nadian:** Conceptualization (equal); Data curation (equal); Formal analysis (equal); Funding acquisition (equal); Investigation (equal); Writing‐original draft (equal); Writing‐review & editing (equal). **Hossein Abbastabar Ahangar:** Conceptualization (equal); Data curation (equal); Formal analysis (equal); Funding acquisition (equal); Investigation (equal); Methodology (equal); Project administration (equal); Supervision (equal); Writing‐original draft (equal); Writing‐review & editing (equal). **Aazam aarabi:** Conceptualization (equal); Investigation (equal); Methodology (equal); Validation (equal); Writing‐original draft (equal); Writing‐review & editing (equal). **Mohammad Hossain Azizi:** Conceptualization (equal); Methodology (equal); Supervision (equal); Writing‐review & editing (equal).

## ETHICAL APPROVAL

The authors declare no conflict of interest. The study was conducted according to the guidelines of the Declaration of Helsinki. The study's protocols and procedures were ethically reviewed and approved by Research Ethics Committees of Islamic Azad University‐ Najafabad Branch (Approved ID IR.IAU.NAJAFABAD.REC.1400.062; IR.IAU.NAJAFABAD.REC.1400.062; 2020–10–29).
